# Defining quantitative rules for identifying influential researchers: Insights from mathematics domain^☆^

**DOI:** 10.1016/j.heliyon.2024.e30318

**Published:** 2024-04-29

**Authors:** Ghulam Mustafa, Abid Rauf, Ahmad Sami Al-Shamayleh, Muhammad Tanvir Afzal, Ali Waqas, Adnan Akhunzada

**Affiliations:** a Department of Computer Science, Univeristy of Engineering and Technology, Taxila, 47080, Punjab, Pakistan; b Department of Data Science and Artificial Intelligence, Faculty of Information Technology, Al-Ahliyya Amman University, Amman, 19328, Jordan; c Department of Computing, Shifa Tameer-e-Millat University, Islamabad, 44000, Pakistan; d Mathematics Foundation Program, University of Doha for Science and Technology, Doha, 122014, Qatar; e College of Computing & IT, University of Doha for Science and Technology, Doha, 122014, Qatar

**Keywords:** Decision tree, Rule mining, Author assessment parameters, Multilayer perceptron, Recursive elimination technique

## Abstract

In the midst of a vast amount of scientific literature, the need for specific rules arise especially when it comes to deciding which impactful researchers should be nominated. These rules are based on measurable quantities that can easily be applied to a researcher's quantitative data. Various search engines, like Google Scholar, Semantic Scholar, Web of Science etc. Are used for recording metadata such as the researcher's total publications, their citations, h-index etc. However, the scientific community has not yet agreed upon a single set of criteria that a researcher has to meet in order to secure a spot on to the list of impactful researchers. In this study, we have provided a comprehensive set of rules for the scientific community within the field of mathematics, derived from the top five quantitative parameters belonging to each category. Within each categorical grouping, we meticulously selected the five most pivotal parameters. This selection process was guided by an importance score, that was derived after assessing its influence on the model's performance in the classification of data pertaining to both awardees and non awardees. To perform the experiment, we focused on the field of mathematics and used a dataset containing 525 individuals who received awards and 525 individuals who did not receive awards. The rules were developed for each parameter category using the Decision Tree Algorithm, which achieved an average accuracy of 70 to 75 percent for identifying awardees in mathematics domains. Moreover, the highest-ranked parameters belonging to each category were successful in elevating over 50 to 55 percent of the award recipients to positions within the top 100 ranked researchers' list. These findings have the potential to serve as a guidance for individual researchers, who aimed on to making it to the esteemed list of distinguished scientists. Additionally, the scientific community can utilize these rules to sift through the roster of researchers for a subjective evaluation, facilitating the recognition and rewarding of exceptional researchers in the field.

## 1 Introduction

Millions of active researchers generate a huge corpus of research articles daily [1], [2], [3]. However, the immediate impact and quality of these articles remain uncertain. It takes time for these articles to receive acknowledgment. Thus, creating a delay for award-winning researchers to impact the scientific community based on their research. The evaluation of the researcher's and its impact has created a big fuss within the scientific literature, resulting in various methods being proposed. Different scientific societies have adopted distinct approaches to measure the impact of the researchers. Despite the ongoing discussions, a consensus has yet to be reached within the scientific community regarding a unified method for recognizing outstanding contributions by scientists. Numerous methods for assessing the researchers impact have been explored in the literature [4][5][6]. One approach involves collecting reviews from domain experts. While this method holds credibility due to the expertise of the professionals of the field but it is a labor-intensive manual process. Another common strategy in use involves quantifying the total number of publications or citations. However, these methods are not without their limitations, as researchers might publish in the lower-quality journals [7] or engage in self-citation, which involves citing their own works [8].

The scientific community experienced a significant change when Hirsch proposed the h-index, a measure that looks at both the quality and the quantity quality of a researcher's work [9][10]. Despite the widespread adoption of this index by numerous researchers, subsequent studies have revealed certain constraints and shortcomings. For instance, Diense points out that obtaining more citations for h-core articles does not necessarily improve the h-index score [11]. Another researcher, [12], explained in a survey that less cited articles are ignored by the h-index, even when they have similar citations to the more-cited ones, and so on. To address these issues, researchers have developed many different methods to measure research impacts [13]. A recent study [12] found that in the literature, there are now over 70 proposed measures, such as A-index [14], AR-index [15], M-Quotient [16], k index [17], etc. Whenever a new method is developed, it is usually tested using different sets of hypothetical situations or by data provided. This makes it difficult to determine which measure is most important among all [18].

In light of the introduction of indices designed to overcome the limitations of the h-index, several studies have put forth evaluations of these newly proposed parameters. In 2016, Dienes assessed the h-index, g-index, and complementary h-index to rank researchers in the field of mathematics [11]. In 2018, De et al. conducted an evaluation of the h-index and its variants in civil engineering, including those based on citation intensity and publication age [19]. Schreiber et al. (2019) evaluated the h-index and its variants using a dataset in neurosciences [20]. In 2019, Ain et al. [18] and Ghani et al. [21] systematically evaluated citation intensity-based indices of the h-index, utilizing a comprehensive dataset from mathematics. Moreira et al. (2021) assessed various indices on a comprehensive dataset in civil engineering, aiming to identify the most effective metrics for evaluating author performance [22]. Recently, Mustafa et al. (2023a, 2023b) evaluated Publication, Citation-based metrics, and publication age-based parameters on a mathematics dataset [23], [24]. Ahmed et al. (2023) [3] evaluated author count-based parameters on the same dataset. Usman et al. 2021 [7] employed logistic regression for ranking author assessment parameters, with a limited parameter number; in their second study, they used a neural network for ranking the same author assessment parameters. Similarly, Ahmed et al. 2023 [6] utilized dynamic random forest with brute optimizer to rank author assessment parameters.

In the present circumstances, to the best of our knowledge, there is an absence of well-defined rules that encompass the abundance of parameters at hand. These rules clarify which parameters should be used and which ones should not be. Moreover, there are no criteria defined for a researcher to set a goal that could make him an impactful researcher. This emphasizes the need to establish clear rules that can guide both newcomers in making significant contributions and the scientific community in to selecting candidates for research awards [25], [26]. Recent studies have attempted to evaluate the impact of parameters (h-index & its variations) on the recipients of research awards [23], [24], [26]. However, these studies considered only a limited number of parameters. Another study [8] proposed some rules; but they were drawn from a restricted set of parameters.

In this study, we formulated rules based on parameters ranked at the top across various categories. We utilized nearly 64 distinct author assessment parameters, which were categorized into four main groups such as, 1) primitive parameters, 2) publication and citation count-based parameters, 3) publication age-based parameters, & 4) Author Count-based Parameters. Given the extensive number of parameters, our initial step involved ranking using a Multilayer Perceptron classifier in conjunction with the Recursive Elimination technique. Through this process, we ascertained the importance score for each parameter, which allowed us to rank the parameters within each category. Subsequently, we select the top five parameters for rule generation. The mining rules were accomplished using a Decision Tree algorithm from Machine Learning. This study holds the potential to significantly aid individual researchers in enhancing their prospects for winning awards in the right direction. Moreover, it provides the scientific community with a means to select the best researchers by quantifying their impacts within their respective fields. For experimentation, we employed a dataset from the domain of mathematics, which is already used in the existing literature. This dataset comprised 525 award-winning researchers and an equivalent number of non-award-winning researchers.

In this study we have addressed the following research questions.

• Which specific quantitative parameters exert the most substantial influence on researchers who have received awards within each individual category?
• Among the categories of parameters considered, which ones exhibit a higher degree of influence in the process of rule generation, thereby leading to broader coverage of awardees?

The finding from this study hold the potential to serve as a foundation upon which the scientific community can establish criteria for awarding scientific honors. Additionally, these findings can also function as guiding principles for fledgling researchers venturing into the field.

The rest of this paper is organized as follows. In literature Review, we present a concise overview of the literature, the Methodology outlines our approach for ranking and establishing quantitative rules for scientific parameters. The Results, presents the results of our study. In the Conclusion section, we present a summary of this research. Lastly in future Work, we will explore the potential areas for future advancements in this domain.

## 2 Literature review

In this era, the scientific community needs to define universal criteria for evaluating researchers' performance to ensure equitable and impartial ranking procedures. Researcher impact is assessed through various metrics, including publication count, citation count, h-index, h-index variation, and a combination of approaches. Scholars have nominated for various initiatives within the academic and professional community through subjective evaluations [27], [28], [29], [30]. However, these traditional approaches primarily rely on metrics, such as publication and citation counts, which are not universally applicable for global assessments. Critiques of these metrics have arisen because of their limitations. Researchers who produce a high volume of publications are often considered active researchers, but this can be misleading if the publications are in low-impact journals or low impact conferences [31]. Similar to this, citation counts are not always reliable indicators of research influence, as they can be influenced by self-citations or negative citations [32]. In response to these shortcomings, the h-index has gained attention for its simplicity; however, it also has its own limitations. For example, it does not necessarily reflect a researcher's impact when citations increase for h-core papers [33]. Also this is not well suited for new researchers who need time to accumulate citations and increase their h-index. Furthermore, it sometimes favors researchers who are currently inactive [34], [35]. To overcome these issues, scientists have proposed numerous alternative parameter to complement the h-index's shortcomings such as A index, k index f index and so on.

With the proposition of new indices, some researchers evaluated (h index & its variants) for finding the impact of these parameters. [13] conducted a study that focused on assessing h-indices. They used award data from mathematical societies, which are considered as a benchmark. In this study, the author identified that the complete h-index outperformed all other indices. In their next study, [26] performed the evaluation of the h index and its variants, which were not discussed in their previous study, and found that the h index outperformed the remaining alternatives. The [36] assessed quantitative parameters within the domain of scientific neuroscience. In their study, they have reported that the hg-index and R-index play a significant role in prominently positioning awardees at the forefront in the researchers list. Similarly, [37] conducted a study to assess quantitative parameters within the mathematics domain. They established correlations between these quantitative parameters, and ranked these parameters based on award-winning researchers. However, they focused on researchers who received awards prior to the introduction of these parameters. As such, the correlation found between awardees and quantitative parameters could be coincidental, as those awardees were not nominated based on indices (h index & its variants). To mitigate this constraint, [7] conducted a study for the assessment of the indices (h index & its variants). This approach is based on the utilization of data from the civil engineering domain. Instead of employing a random selection process, they opted for a targeted approach. They specifically selected individuals who had received awards and those who had not within the same temporal span. This selection was exclusive to researchers who had been honored by prestigious civil engineering scientific societies since 2005. However, their dataset is not sufficiently comprehensive to definitively determine the importance of parameters for award winners. Furthermore, the current literature lacks clearly defined criteria for these parameters, which researchers can use as a foundational benchmark for subjective conduction evaluation. Furthermore, one of the study conducted by [8], generated a rule for scientific community but they chose a top parameter for defining rules from a limited no. of total parameter. Recently, [23], [24] conducted two studies, in first they have evaluated publication and citation count based parameter in which they found that normalized h index outperformed all the remaining parameters and in second they have evaluated publication age based parameter in which they found that Ar index outperformed the remaining indices. For experimental purposes, they used a mathematical domain dataset.

After conducting a comprehensive literature review, we studied a number of proposed techniques and conducted evaluation surveys of these parameters. From these studies, our findings indicate that approximately a decade ago, researchers were evaluated based on their publications and citations. However, the approach shifted in the following decade, with a focus on using variations of the h-index without accounting for study limitations or context. When novel methodologies are introduced, they often emerge in unconventional ways or are tested using diverse datasets. Due to this divergence in data sources and validation methods, assessing the individual indices importance is challenging. Thus, it is crucial to thoroughly evaluate the indices when deriving rules that incorporate individual parameters or parameter combinations. This enables the distinction between prolific and nonprolific researchers. To formulate these rules, it is necessary to understand the patterns of award recipients. These rules could potentially elevate non awardees by including them in the pool of awardees.

## 3 Methodology

**Figure 1:**
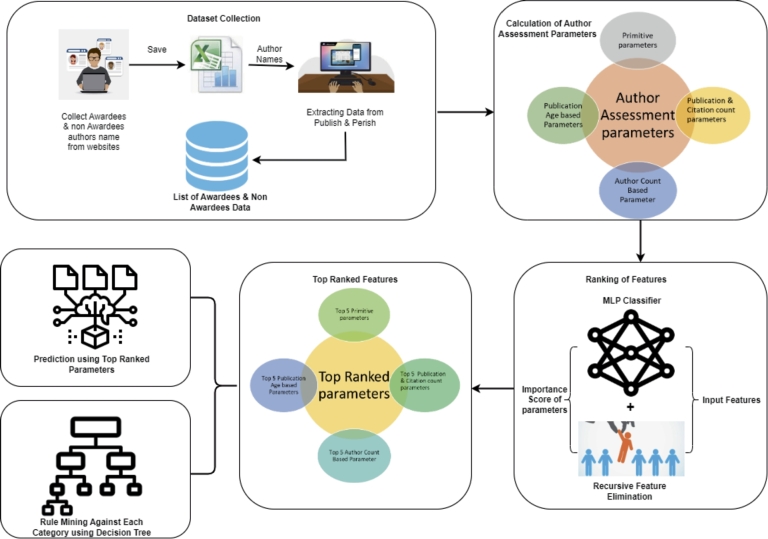
The architecture diagram represents multiple phases in the proposed system. The dataset collection phase illustrates the process of gathering data, followed by the depiction of parameter categories in the next phase. Subsequently, ranking techniques are represented, and the subsequent phases portray the prediction of awardees based on the top-ranked parameters and the generation of rules for each category.

After a critical analysis of the literature, we will now discuss rule mining for researchers belonging to the domain of mathematics. We employed a multilayer perceptron classifier with a recursive elimination technique to find the most significant quantitative parameters for each category based on the abundance of parameters. Fig. 1 represents the architecture diagram containing different steps, such as (i) Domain Selection & Dataset Collection, (ii) Calculation of Author Assessment Parameters, (iii) Ranking of Author Assessment Parameters, (iv) prediction of awardees using top-ranked parameters, & (v) rule mining using the top five Parameters against Each Category.

In the following sections, we explain all of the above points.

### 3.1 Domain selection and dataset collection

**Figure 2:**
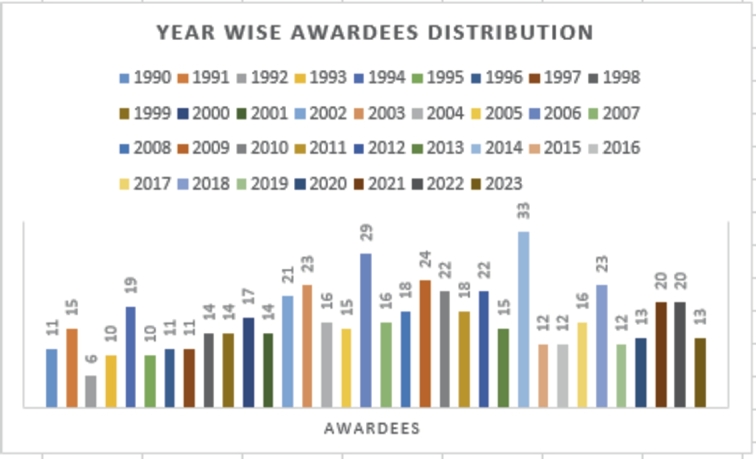
Present the count of awardees year-wise, with each bar representing the number of awardees for a specific year.

For experimentation, a dataset specific to a particular domain was required. In this study, the Mathematics domain was selected as the main focus. That too, due to its extensive history and significant contributions in research. Which makes it the optimal choice for evaluating the proposed methodology. Moreover, within this domain, various scientific societies bestowed annual awards to distinguished researchers, that were based on the impact of their research work. Due to the comprehensiveness of this domain, several studies have already opted for its utilization [18], [23], [24], [38]. Within this domain, we selected the dataset used by [23], [24]. This dataset comprised 1050 records, encompassing information about both awardees and non-awardees. Specifically, the dataset contains 525 non-awardees and 525 awardees. For the awardees' data, a list of 30 internationally recognized awards, along with their significant importance (within the mathematical community) were compiled. These awards are esteemed accomplishments for mathematicians and researchers were awarded by renowned mathematical societies such as, the London Mathematical Society (LMS),^1^ International Mathematical Union (IMU),^2^ Norwegian Academy of Science and Letters (NASL),^3^ and the American Mathematical Society (AMS).^4^ The proportion of data related to non-awardees in the dataset was derived from [18] & [21]. However, since the original datasets used by [18] & [21] contained only a limited number of awardees entries, extending until 2013, [23]. expanded this dataset by gathering updated information on awardees up to 2023. The detail statistics of dataset is given in Table 1. For the collection of awardees' data, various society websites (LMS, IMU, NASL, and AMS) within the mathematics domain were visited to gather names and corresponding years of awards given to researchers over the past three decades. The distribution of awards across years is presented in Fig. 2. To extract data on both awardees and non-awardees, the Publish or Perish^5^ platform was utilized, employing a hold-on strategy that enabled the collection of researchers' data even before their award-receiving years. This tool utilized a sophisticated algorithm to retrieve primary data along with author metadata from Google Scholar. In order to ensure equity within the dataset, data on non-awardees was collected in the same proportion of the number of awardees for each year. For instance, if there were 15 awardees in 1991, data from the 15 non-awardees prior to 1991 were collected using similar techniques. Before conducting any analysis or evaluation, thorough data cleansing from sources such as Google Scholar was essential. This process aims to eliminate incorrect or irrelevant information, commonly referred to as noise, which could compromise the accuracy of the results. The data cleansing comprises several steps, including verification of data accuracy and removal of duplicate entries. In our extensive research dataset, two crucial processes were performed to enhance the quality and relevance of the data. First, a filter is applied to make sure the consistency of each research article within the mathematical field, removing any irrelevant content. This step aided in refining the dataset to concentrate solely on the relevant domain. Subsequently, an author disambiguation process was executed to find and remove duplicate entries resulting from researchers publishing using different variation of names.

**Table 1:** Dataset Statistics.

Dataset Statistics	
Total Authors	1050 (525 Awardees / 525 Non Awardees)
Citation Count	14,369,007
Publication Count	204,796
**Benchmark Dataset Awardees**	
Total Awardees	525
Total Awardees belong to AMS	257
Total Awardees belong to IMU	59
Total Awardees belong to LMS	188
Total Awardees belong to NASL	21

### 3.2 Calculation of author assessment parameters

**Figure 3:**
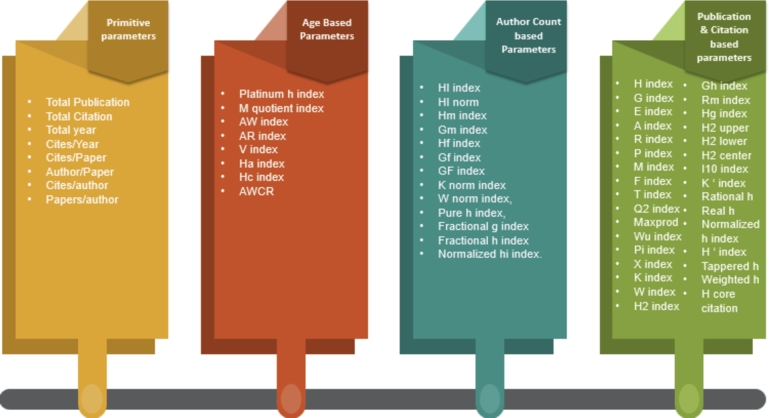
Parameter Categories, with the names of parameters listed under each respective category.

In this section, we calculate the Sixty-four author assessment parameter belonging to four different categories on the collected dataset. The list of the categories with respective indices is presented in Fig. 3.

Moreover, the formulas of these indices with explanation are presented in Table 3 (Appendix A).

### 3.3 Ranking of author assessment parameters

**Figure 4:**
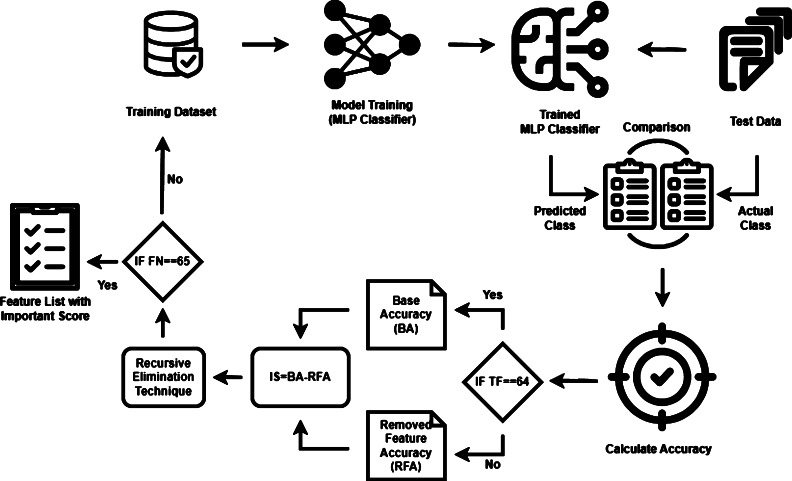
Feature Ranking process, the dataset was initially divided into a 20:80 ratio. A MLC was trained, and a validation sample was provided to the trained model. The accuracy achieved during this prediction stage was considered the Baseline Accuracy (BA) when Total feature (TF) is equal to sixty four. In the next iteration, the feature removal process began. One parameter was removed from the feature list, and the model was trained on the updated feature set. The new accuracy (RFA) obtained from test sample prediction was then subtracted from the BA, gives Importance Score (IS) against removed feature. The entire procedure was iterated for each parameter in the dataset. Upon iterating through all features, when the Feature Number (FN) reached sixty-five, it signified the completion of iterating through all features. The algorithm generated two lists. The first list contains the names of the features, while the second list contains their corresponding importance scores. Based on these importance scores, the parameters were sorted, providing us with a parameter ranking.

In the realm of machine learning, the task of feature ranking holds significant importance. It plays a pivotal role in discerning the most influential features used for a variety of purposes [39]. These purposes encompass dimensionality reduction, enhancing interpretability, mitigating overfitting, optimizing prediction processes, and refining Feature Engineering. The overall process of feature ranking is depicted in Fig. 4. To assess the importance of these features, we employed a multilayer perceptron classifier (MLP) in conjunction with a recursive elimination technique. The MLP classifier is widely recognized in the literature for its versatility, serving as a fundamental algorithm in various problem domains, including classification, prediction, regression, among others [40]. This classifier is constructed as a feed-forward artificial neural network, consisting of multiple hidden layers [41]. In classification-related tasks, the number of features aligns with the number of neurons in the input layer, while the total number of classes used for data classification corresponds to the number of neurons in the output layer. Intermediate layers, situated between the input and output layers, form a fully connected network trained through the backpropagation algorithm. During the forward propagation phase, the network computes the output for each layer using an activation function based on the previous layer's output, combined with the respective weight and bias values presented in Equation (1).

(1) X=WA+b

Where X represent output matrix, W represent weight matrix and b represent bias vector.

To guarantee that the MLP's output falls within a predefined range, an activation function is applied. This function serves to normalize the output of each layer, ensuring that it conforms to the desired range, as illustrated in Equation (2).

(2) C=g(X)

Where C represent the activated output matrix.

In this study, we used activation function name as Rectified Linear Unit (ReLU) for the hidden layers and Softmax activation function for the final output layer. The activation function, is defined by Equation (3), acts by setting values less than zero to zero while preserving positive values. This introduces essential nonlinearity into the network, enhancing its capacity to learn complex patterns.

(3) f(X)=max(0,X)

In Equation (4) softmax function is defined which is a well-established choice for classification problems. It mitigates the limitations of the sigmoid function and ensures that the probabilities assigned to each class in the output layer sum up to one. By applying Softmax, we can identify the most probable prediction for a given set of inputs.

(4) $$Softmax(X_{i})=\frac{e^{X_{i}}}{\sum_{i=1}^{J}e^{X_{i}}}$$

Where J represent the number of classes and Xi denote the ith output value.

Furthermore, loss function is represented in Equation (5), which is used to quantify the disparity between predicted and actual values within the MLP. It stands as a pivotal metric for evaluating the model's performance. The loss function's computation enables the assessment of the degree of mismatch between the predicted outcomes and the desired target values. Following this assessment, a backpropagation algorithm is employed to iteratively adapt the weights (w) and biases (b) within the neural network. This iterative process is instrumental in optimizing the model's performance by minimizing the loss. Through this fine-tuning of parameters, the network is progressively refined to enhance its ability to make accurate predictions.

(5) $$L(z,\overset{ˆ}{z})=\frac{1}{n}\sum_{i=1}^{n}{\left(z_{i}-\overset{ˆ}{z_{i}}\right)}^{2}$$

Where n denotes number of records and $z_{i}$ represent the predicted value while $\left(z_{i}\right)$ represent the actual value. Furthermore, whenever we are using deep-learning models, it is of paramount importance to mitigate the risk of overfitting. When a deep neural network possesses an excessive number of layers, it can give rise to issues like gradient vanishing or explosion, severely hampering the model's performance and exacerbating overfitting. To address these challenges, a technique known as batch normalization was introduced by [42]. The primary objective of batch normalization, as outlined in Equations (6), is to counteract gradient explosions or vanishing. This is accomplished by normalizing the output values after each hidden layer, ensuring that they remain within reasonable bounds and do not become excessively large or small. The batch normalization process involves calculating the difference between each output value and the mean value of the entire vector, and subsequently dividing this difference by the standard deviation. In our MLP model, batch normalization was thoughtfully incorporated after each hidden layer to effectively combat overfitting, thereby enhancing the model's generalization capabilities.

(6) $$X_{i}=\frac{X_{i}-Mean_{i}}{Standard\;Deviation_{i}}$$

Where $X_{i}$ represent $i^{t}h$ hidden layer output matrix and $Mean_{i}$ represent mean value of $X_{i}$.

In this study, 10 hidden layers in MLP Classifier. The ReLU activation function was employed in each hidden layer, which consisted of 10 neurons. The count of hidden layer and neurons was selected based on multiple experiments. To normalize the range of values, batch normalization was applied after each hidden layer. The selected features were input into the neural network by using input layer. Subsequently, model was trained using forward & backward propagation techniques, whereas the output layer employed the Softmax activation function to generate class probabilities. During the prediction phase, a vector is produced containing class probabilities, and argmax function (described in Equation (7)) was used to identify the highest probability value and return its corresponding index.

(7) Ouptut=max(PredictedVector)

For training our model, we used the Adam optimization algorithm, known for its dynamic adjustment of the learning rate based on recent weight gradients. In our configuration, we set the learning rate to 0.0003, used a batch size of 64, and conducted training for a total of 100 epochs. To prevent overfitting, we thoughtfully implemented the early stopping technique. This technique monitors the training process and halting it when early signs of overfitting become apparent, thereby preserving the best model parameters. In our setup, we configured the early stopping parameter to 40, indicating that if the loss on the validation set failed to decrease for more than 40 consecutive epochs, it signaled that the model had indeed overfit. At this juncture, the training process was promptly terminated, and any changes made to the model's parameters during those epochs were reverted, ensuring the model's generalization capacity was preserved.

Furthermore, with this classification model we have employed Recursive elimination technique with some modification for the calculation of importance score. This technique is mostly used to identify relevant features that contribute significantly to model performance [43]. Furthermore, this technique reduces the dimensionality of the dataset and improves the model interpretability, efficiency, and generalization ability [44]. RET iteratively removes irrelevant or redundant features and focuses on a subset of features that have the most significant impact on the overall model performance. In this study, the first step involves dividing the dataset into a training set and validation set using 20:80 ratios. The training dataset was used to train a multilayer perceptron classifier for classification purposes (discussed earlier in this section). Subsequently, a validation sample was provided to the trained model, and the model predicted the class label for each sample. The accuracy achieved during this prediction stage was considered the baseline accuracy when all the features were included. The next phase of the technique focuses on feature removal. One parameter was removed from the feature list, and the dataset was again divided into training and validation sets. The multilayer perceptron classifier is then trained using the updated feature set. Following training, a test sample was utilized to predict the class label, and the accuracy was noted. The new accuracy obtained was subtracted from the baseline accuracy, yielding a subtraction result that served as the importance score for the removed feature. This process was repeated for each parameter with at least five different epoch phases, yielding an importance score. Equation (8) represents the importance score calculation.

(8) $$F_{IS}=\frac{1}{5}\sum_{i+=20}^{100}(BLA_{i}-WOPA_{i})$$

Where $F_{IS}$ represent feature important score, $BLA_{i}$ represent the baseline accuracy against in $i^{th}$ iteration and $WOPA_{i}$ represent the without parameter accuracy of the $i^{th}$ iteration.

The entire process was repeated for each parameter in the dataset. After iterating through all the features, the algorithm generates two lists. The first list contains the names of the features, whereas the second list contains the corresponding importance scores. On the basis of this importance score, the parameters were sorted based on their respective scores. This sorting process provides us with parameter ranking.

### 3.4 Prediction using top ranked parameters

After ranking we have Selected the top five parameters from each category (Primitive parameter, Publication and citation count based parameter, Author count based parameter, Publication age based parameter). Furthermore, by using these parameters we have to check the awardees trend in a ranked list of top 100 researchers records. These results will show the potentiality of top parameters against each category.

### 3.5 Rules mining using top 5 parameters against each category

In machine learning, the decision tree is widely recognized as a go-to choice for a variety of tasks, including prediction, classification, and rule mining [45], [46]. Due to their popularity in this task, we employed decision trees due to its intrinsically interpretable nature and easy to implement.

Moreover, process of building a decision tree commences with the division of the source dataset into two subsets based on a chosen attribute. This division initiates a recursive learning procedure, where each subtree learns from the subsequent subtree. This recursive learning continues until one of two conditions is met: either a subset containing data from only one class is reached, or further subdivision of the data into subtrees ceases to impact the learning process. Decision trees are adept at generalizing, detecting patterns, and categorizing data within a dataset through the application of statistical methods. They are particularly valuable for exploratory tasks because they do not necessitate specific prior knowledge or criteria for construction. Decision trees are renowned for their high accuracy and are considered a standard in the realm of classification tasks.

The process of splitting decision tree nodes is typically guided by attributes. These attributes are either Gini-index or information gain. Due to nature of the dataset, we have employed the Gini-index which is mostly preferable with numeric type data [47]. Moreover, this decision is driven by the fact that information gain is commonly employed to identify the most informative attributes, which can be less suitable in cases which contain numerous different attribute values [48]. In contrast, the Gini-index is a statistical measure used to assess impurities within data [47]. Impurity is quantified as the number of data points that are incorrectly classified when all elements belong to the same class. The Equation (9) represents Gini-formula.

(9) $$GI=1-\sum_{j=1}^{n}{\left(P_{j}\right)}^{2}$$

Where $P_{j}$ represent object probability in the dataset being classified into class j. Moreover, it takes values between 0 and 1, and its interpretation is as follows:

• A Gini index of 0 indicates perfect purity, where all elements in the node belong to the same class.
• A Gini index of 1 implies maximum impurity, meaning the elements are evenly distributed among the classes, and there is no predominant class.

In addition, our dataset encompasses a substantial number of author assessment parameters, resulting in a decision tree that tends to be overly intricate and dense. To alleviate this complexity and eliminate extraneous rules, we employed a post-pruning technique. Post-pruning entails the initial growth of the tree to its full extent, followed by the removal of nodes that contribute negligibly to the overall performance of the tree. This method involves the calculation of a cost parameter for each subtree considering both its accuracy and complexity, quantified by the number of nodes it contains. Subtrees with higher cost values were pruned, resulting in a more streamlined and generalizable tree. This refined tree allows for the extraction of generalized and well-suited rules for each category parameter.

## 4 Results discussion

Now, in this section we have explained our experimental results obtained from proposed technique by using mathematics dataset.

### 4.1 Parameters ranking

Moving forward, we have displayed a list of the most effective parameters obtained using RFE technique along MLP. Subsequently, these high-performance parameters are utilized in the crafting rules for the mathematical domain using a decision tree.

#### 4.1.1 Primitive parameters ranking

**Figure 5:**
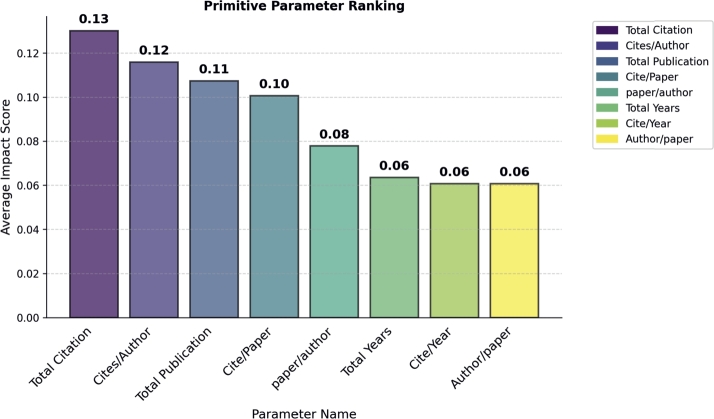
Primitive Parameter Ranking.

In the primitive parameters category, we found that the total citation parameter had a higher impact (Importance Score:0.13) on model performance than all the other parameters (see in Fig. 5). The cite/author and total publications take the second and third positions by achieving importance score 0.11 and 0.10 respectively. In order to assess how the total citation count parameter influences the selection of award recipients within the field of mathematics, we conducted a classification of individuals who received awards and those who did not. The total citation individually, achieved 62% percent accuracy in classification of awardees, while the second position cite/author achieved 50% and the third position total publication 47%. The accuracies of the remaining parameters were less than 40. Thus, if you increase your total citations, the chances of your award winner increase.

#### 4.1.2 Author count based parameters ranking

**Figure 6:**
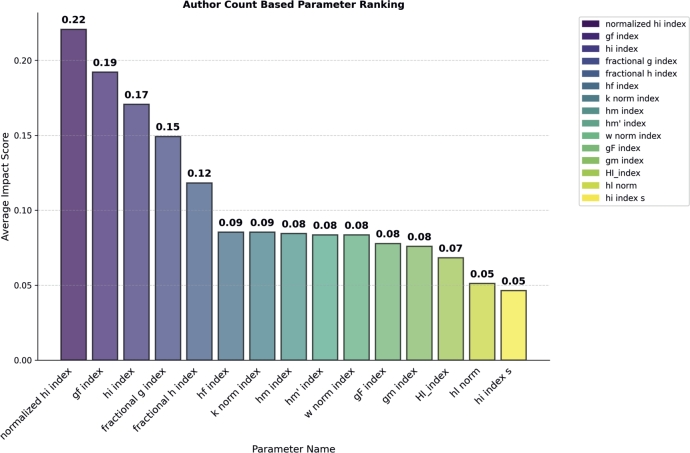
Author Count based Parameter Ranking.

In the Author Count-Based Parameters category, we found that the normalized hi index outperformed all other parameters by creating a huge impact on model performance by achieving an Importance Score of 0.22 (see in Fig. 6). The gf index and hi index take the second and third positions, respectively, by achieving importance score 0.19 and 0.17 respectively. Similar to the above category, for the verification of normalized hi index, we performed classification of awardees and non-awardees. The normalized hi index achieved 71% accuracy, the gf index achieved 68%, and the hi index achieved 55% accuracy. The remaining parameters in this category achieved an accuracy of less than 45%.

#### 4.1.3 Publication age based parameters

**Figure 7:**
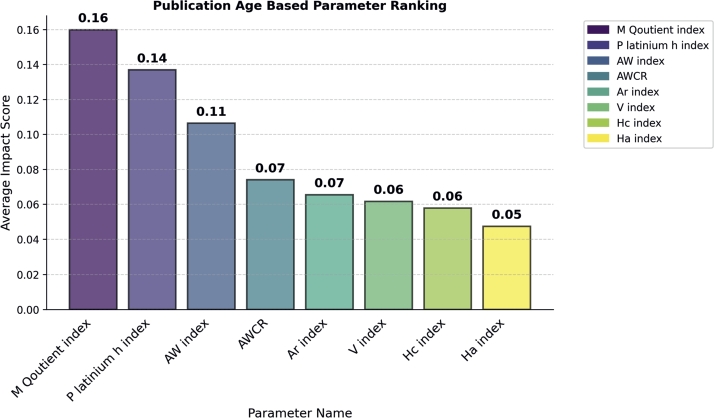
Publication Age Parameter Ranking.

In the Publication Age-based Parameters category, we found that the m-quotient outperforms all the other parameters by impacting model performance by achieving an Importance Score of 0.16 (see in Fig. 7). The platinum h and Aw indices take the second and third positions, respectively, by achieving importance score 0.13 of 0.10 respectively. Similar to the above category, for verification we performed classification of awardees and non-awardees. The m-quotient individually achieved 60% accuracy in classification of awardees, while platinum h index achieved 51%, Aw index achieved 44%, and the remaining one achieved less than 35%.

#### 4.1.4 Publication and citation count based parameter

**Figure 8:**
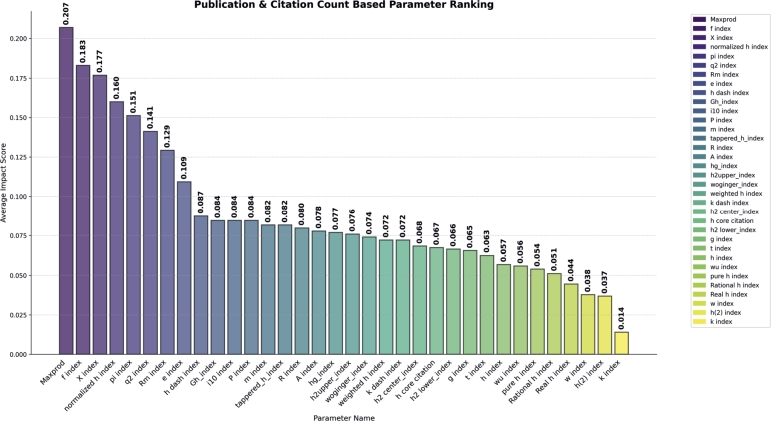
Publication and Citation Count based Parameter Ranking.

In the Publication and citation count-based parameter category, we found that Maxprod outperformed all the other parameters by achieving an Importance Score of 0.20 (see in Fig. 8). The f and X indices achieved the second and third positions by achieving importance score 0.18 of 0.17 respectively. Similar to other categories, for verification we performed classification awardees and non-awardees. Maxprod individually achieved 66% accuracy in classification of awardees, while the platinum f index achieved 63%, the Aw index achieved 60%, and the remaining one achieved less than 50%. From this analysis, we concluded that parameters belonging to this category have high performance in classification compared to other categories.

#### 4.1.5 Combination of all parameter

**Figure 9:**
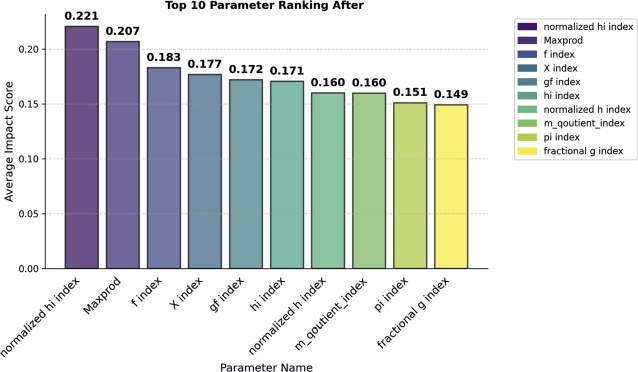
Combine All Category Parameters.

In this section, we combine all the category parameters. When combining all the category parameters, the total count is increased, which is not betterly visualized in one graph; therefore, we only show the top 10 best parameters in the Fig. 9. From this Fig. 9, we have determined that the normalized hi index outperforms all other category parameters. The Maxprod index was placed in the second position and the f index was placed in the third position.

## 5 Rule mining

**Figure 10:**
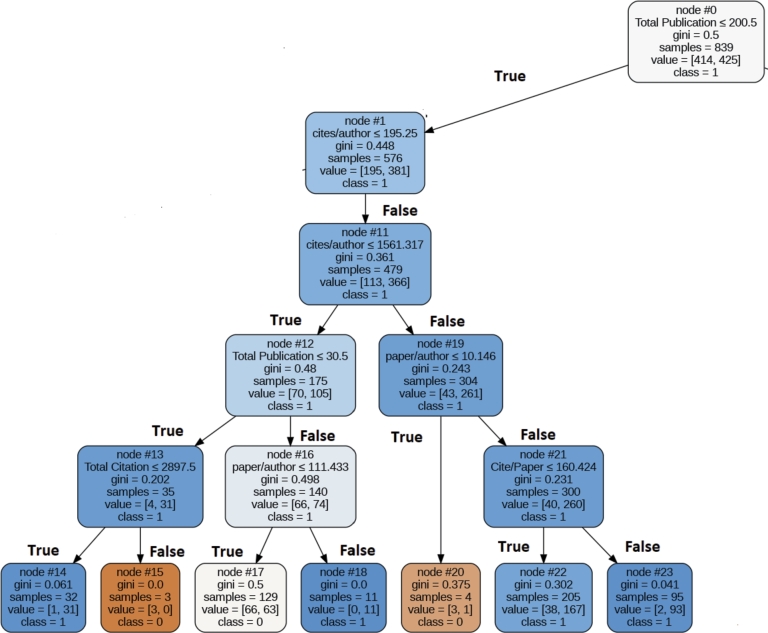
Decision Tree for Extracted Rule for Primitive Parameter.

**Figure 11:**
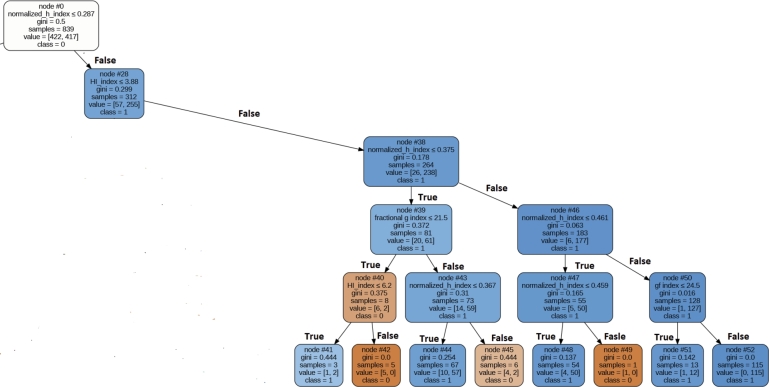
Decision Tree for Extracted Rule for Author Count Based Category.

**Figure 12:**
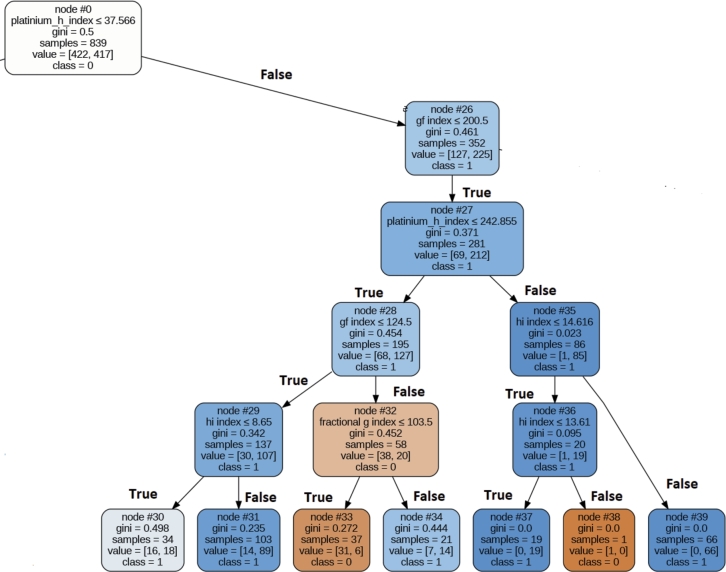
Decision Tree for Extracted Rule for Age Based Category.

**Figure 13:**
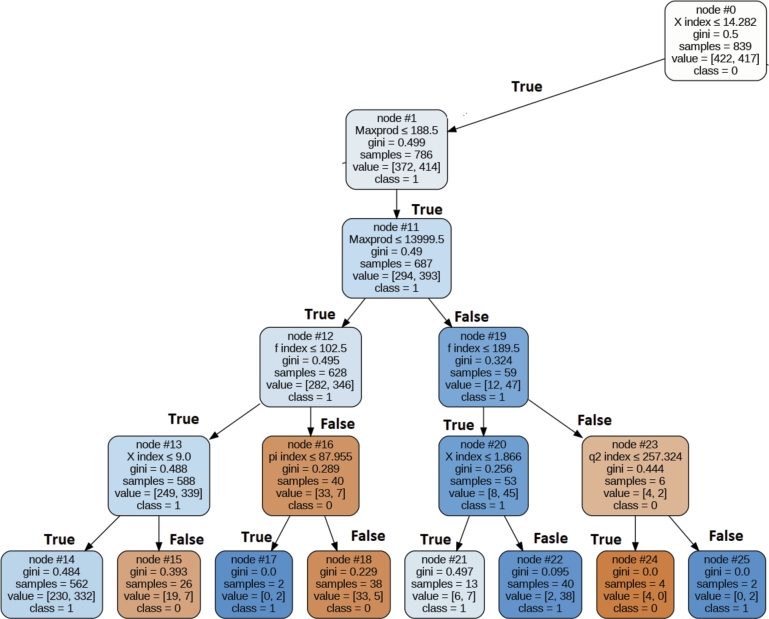
Decision Tree for Extracted Rule for Publication and Citation Count Based Category.

**Figure 14:**
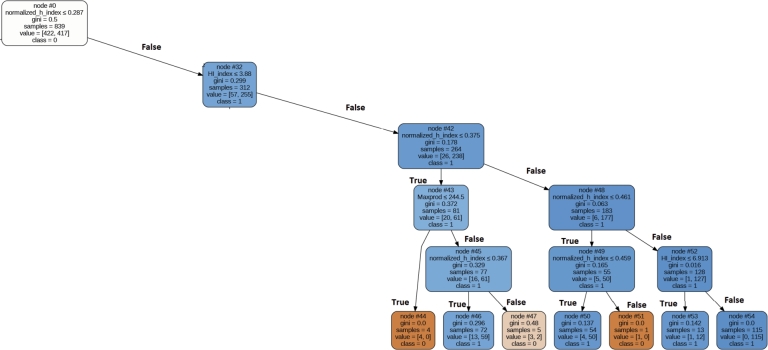
Decision Tree for Extracted Rule for Combining All Category Parameter.

In this section, we introduce a rule for each category based on the top five parameters within that specific category. Figure 10, Figure 11, Figure 12, Figure 13, Figure 14 depict the rules with the highest coverage for each parameter category. These figures were extracted from a complete decision tree against the dataset which contains multiple rules. However, we specifically mined rules that focus only on those aspects we wish to highlight to the scientific community. These highlighted rules hold significant importance in the ranking of the authors.

### 5.1 Rule for the primitive parameters

In the primitive parameters the top five ranked parameters are: Total Publication, Total Citation cite/paper, cite/author, and paper/author. To extract the prevailing pattern among most awardees, we have employed decision tree with Gini measure (Spliting Purpose). This decision tree attained an accuracy rate of 78% for correctly identifying awardees. Fig. 10 illustrates the decision tree representing the extracted rules for awardees. The top rule, which encompasses 55% of awardees in the primitive category, is presented as follows:

IF Total Publication<=200 AND Cite/Author > 195.25 AND Paper/Author<=10 –> Award Recipient

### 5.2 Rule for author count based parameters

In the author count-based category, the top five ranked parameters were as follows: normalized h index, gf index, hi index, fractional h index, and fractional g index. To uncover the prevailing pattern among most awardees, we have employed decision tree with Gini measure (Spliting Purpose). This decision tree achieved an accuracy rate of 73% for correctly identifying award recipients. Fig. 11 visually represents the decision tree outlining the extracted rules for awardees. The top most rule, which encompasses 56% of awardees within the Author Count based category, is detailed as follows:

IF (0.45=>Normalized h index >=0.28) And hi index>=3.88 AND fractional g index>21.4 –> Award Recipient

### 5.3 Rule for age based parameters

In the Age-based Category, the top five ranked parameters are as follows: Platinum h index, Aw index, M Quotient index, Ar index, and AWCR. To uncover the prevailing pattern among most awardees, we employed a decision tree with Gini measure (Splitting criteria). This decision tree achieved an accuracy rate of 68% in correctly identifying awardees. Fig. 12 provides a visual representation of the decision tree outlining the extracted rules for the awardees. The top most rule, which covers 41% of the awardees within the Age-based category, is detailed as follows:

IF (216.9=>Platinium h index >= 37.5) AND Aw index >=208.099 AND M quotient index >=1.3 –> Award Recipient

### 5.4 Rule for publication and citation count based parameters

In this Category, the top five ranked parameters were as follows: F index, Maxprod, pi index, X index, and Q2 index. To uncover the prevailing pattern among most awardees, we employed a decision tree with Gini measure (Splitting criteria). This decision tree achieved an accuracy rate of 71% for correctly identifying awardees. Fig. 13 visually represents the decision tree outlining the extracted rules for the awardees. The top most rule, which encompasses 90% of the award recipients within the Publication and Citation Count-based category, is detailed as follows:

IF (9=> X index <=14.2) AND Maxprod<=188 AND (189=< f index <=102)–> Award Recipient

### 5.5 Combine all parameter

We have now combined parameters from all categories, and among these parameters, the top five ranked ones are as follows: normalized h index, F index, Maxprod, X index, and Hi index. To uncover the predominant pattern among most awardees, a decision tree using the Gini measure as the splitting criterion was employed. This decision tree achieved an accuracy rate of 78% for correctly identifying award recipients. Fig. 14 provides a visual representation of the decision tree that outlines the extracted rules for the awardees. The top most rule, which covers 53% of the awardees are given below:

IF (0.4 =>Normalized h index >=0.287) AND (6.9=>HI index >=3.88) AND Maxprod>=244–> Award Recipient

### 5.6 Rules coverage comparison

**Figure 15:**
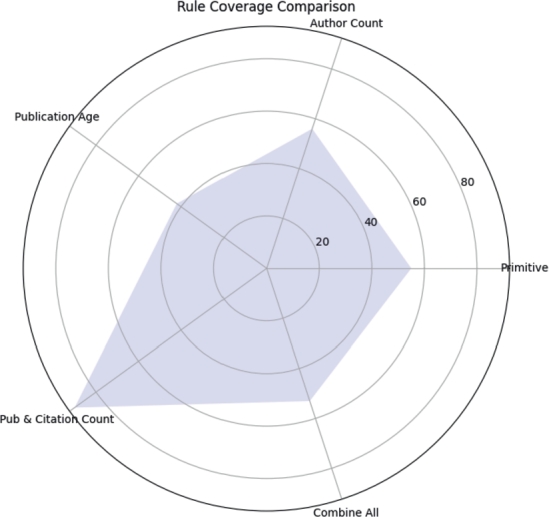
Rule Coverage Comparison.

After creating rules for all categories in this section, we compared the rule coverage between them, as shown in Fig. 15. From the Fig. 15, we have identified that the rule generated using the publication and citation count-based category outperforms all the category rules by achieving 90% coverage, which means that 90% of awardees statistics in the dataset are exactly according to this rule. From this high coverage, we have determined that you can use the publication and citation count-based parameters in combine manner for ranking authors due to their high performance.

### 5.7 Rules validation

To validate the generated rules, we selected a sample from the dataset comprising the awardees from the last three years (2023, 2022, and 2021), totaling fifty-three individuals. Subsequently, we applied each search and mapping to every rule across various parameter categories, determining the correct prediction ratio for each rule. The results are presented in Table 2.

**Table 2:** Rules Validation Percentage.

Parameter Category	Total Awardees	Percentage
Primitive parameters	53	48%
Author count based parameters	53	52%
Rule for Age based parameters	53	43%
Publication and citation count based parameters	53	78%

Analysis of the results revealed certain recurring patterns, notably that rules based on publication and citation counts outperformed all other category rules, correctly predicting 78 percent of awardees.

## 6 Awardees trends in top 100 researchers list

**Figure 16:**
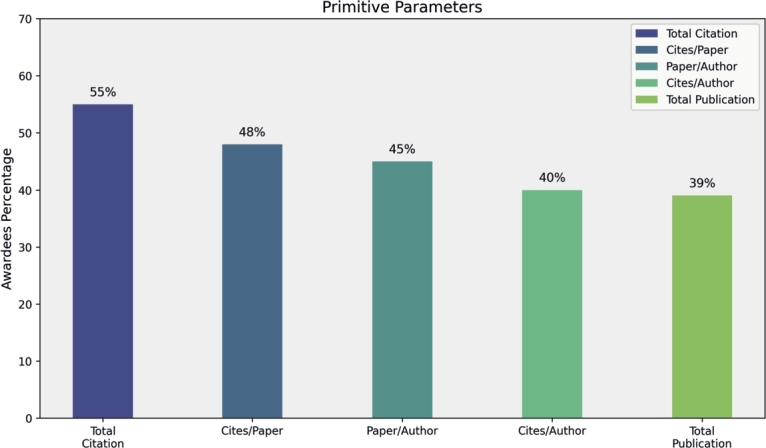
Awardees Percentage for primitive parameters.

**Figure 17:**
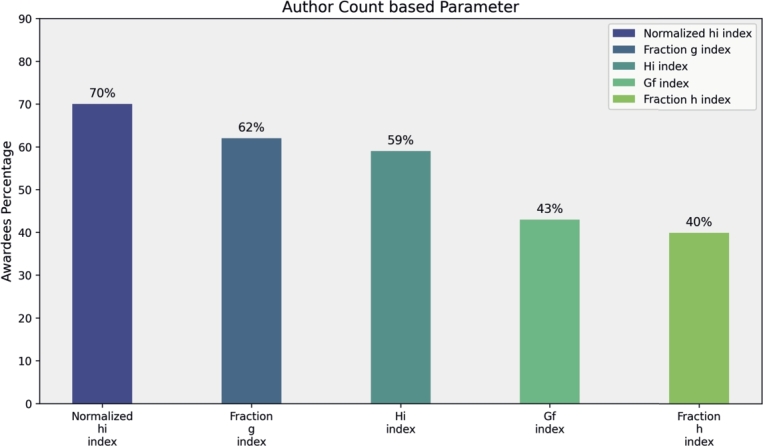
Awardees Percentage for Author Count based parameters.

**Figure 18:**
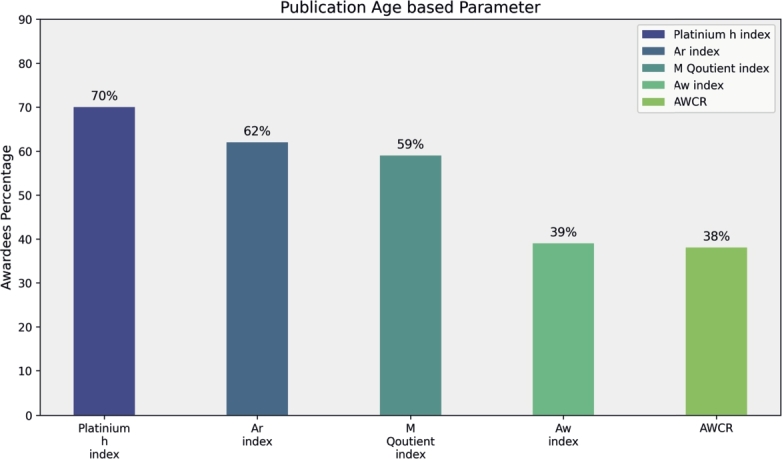
Awardees Percentage for publication Age based parameters.

**Figure 19:**
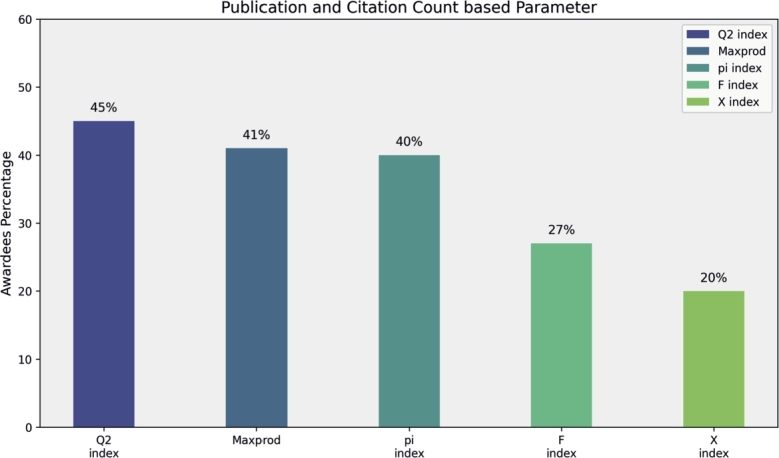
Awardees Percentage for publication and Citation count based parameters.

**Figure 20:**
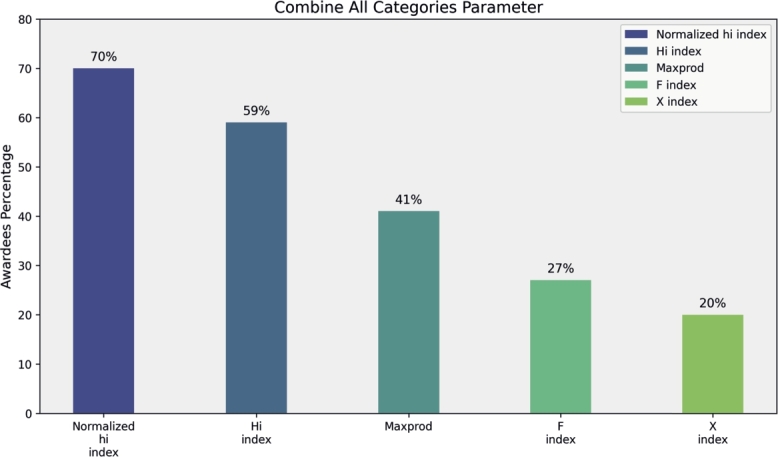
Awardees Percentage after Combine all parameters.

In this section, we analyze award trends among the top 100 researchers by condensing the top five parameters from each of the four categories. Primitive parameters, such as Total Publications, Total Citations, Cites per Paper, Papers per Author and Citations per Author, show that total citations significantly contribute to 55% of awardees' rankings in mathematics fields, while Cites per Paper, Papers per Author, and Citations per Author contribute 48%, 45%, and 40%, respectively (see Fig. 16). Author count-based parameters, including Normalized h-Index and Fractional g-Index, recognize 70% and 62% of researchers, respectively (see Fig. 17). Publication Age-based parameters, such as Platinum h-Index, demonstrate awards for 70% of researchers, while AWCR identifies 38% (see Fig. 18). Parameters based on Publication and Citation counts, like Q2 Index, Maxprod, and Pi Index, acknowledge 45%, 41%, and 40% of researchers, respectively (see Fig. 19). Combining all parameters reveals that Normalized h-Index has the highest impact, recognizing 70% of awardees, followed by h-Index (59%), Maxprod (41%), F Index (27%), and X Index (20%) (see Fig. 20). Author count-based parameters emerge as dominant in elevating awardees compared to other categories.

## 7 Conclusion

The assessment of scientists' achievements has become increasingly important over the past two decades. To evaluate authors' contributions, various unique parameters have been proposed in the literature. This study ranks these parameters, which fall into four distinct categories. The findings reveal that within the first category, total citation counts hold significant relevance. In the Author Count-Based category, the gf index stands out as a strong indicator. Meanwhile, the Publication and Citation Count-Based category highlights the importance of the M Quotient parameter, and in the Publication Age-Based category, the Maxprod parameter is closely associated with mathematics awardees. The study also uses machine learning techniques to analyze the quantitative patterns of prolific researchers, which may have contributed to their inclusion on the list of award recipients. While these patterns can increase the likelihood of making it onto the prolific list, inclusion ultimately depends on subjective evaluation, and there are no guarantees. Additionally, the rule generated using the publication and citation count parameters produced more promising results compared to other category parameters. Consequently, the study suggests that combining publication and citation count-based parameters is more effective for author ranking than relying solely on other categories. The findings of this study offer valuable guidance for individual researchers aiming to be recognized as distinguished scientists. Moreover, the scientific community can use these rules to evaluate and identify exceptional researchers, facilitating the recognition and rewarding of outstanding contributions in the field.

## 8 Future work

In future work, we plan to expand our research from multiple perspectives. Firstly, we aim to evaluate the identified set of parameters across various domains, including Computer Science, Civil Engineering, Neuroscience, and others. This assessment will provide insights into the applicability and effectiveness of our proposed parameters in diverse contexts. Secondly, we intend to introduce new indices that encompass the lifetime achievements of authors. These indices will consider factors like publication count, citation count, and co-author prestige, which are not currently accounted for by existing parameters. Thirdly, we will incorporate Deep Learning techniques to develop a framework for identifying influential researchers. This advanced approach will enhance the accuracy and efficiency of our methodology. Furthermore, beyond the initially chosen parameters, there are additional factors that could contribute to the comprehensiveness of our work. One noteworthy factor is the recently introduced Q-factor, as outlined by (Sinatra et al., 2016). However, determining this diversified parameter goes beyond publications and citations; it also considers aspects such as time, subjective performance in project contribution, dissertation supervision, funding, and more. Our future objective is to construct a dataset that includes subjective factors, thereby broadening the scope of our analysis. Moreover, in upcoming research, we plan to address several inquiries. These include assessing whether the integration of quantitative parameters genuinely enhances the field of Scientometrics, evaluating their practicality for implementation in digital libraries, and exploring the need for new parameters that align with subjective decision-making processes.

## Funding

This Research is funded by 10.13039/100019779Qatar National Library.

## Ethics declarations

Informed consent was unnecessary for this study as it relied on data sourced from public databases. The research excluded any involvement in animal and human experiments, along with other data pertaining to human privacy.

## CRediT authorship contribution statement

**Ghulam Mustafa:** Writing – original draft, Validation, Methodology, Investigation, Formal analysis, Conceptualization. **Abid Rauf:** Writing – review & editing, Validation, Supervision, Methodology, Formal analysis, Conceptualization. **Ahmad Sami Al-Shamayleh:** Writing – review & editing, Validation, Resources, Funding acquisition. **Muhammad Tanvir Afzal:** Writing – review & editing, Validation, Supervision, Methodology, Investigation, Formal analysis, Conceptualization. **Ali Waqas:** Writing – review & editing, Validation, Resources, Funding acquisition. **Adnan Akhunzada:** Writing – review & editing, Validation, Resources, Funding acquisition.

## Declaration of Competing Interest

The authors declare that they have no known competing financial interests or personal relationships that could have appeared to influence the work reported in this paper.

## Figures and Tables

**Table 3:** Indices Calculation Formulas.

Name of Index	Calculation
Total Publication	Total Number of Publications of a Researcher
Total Citation	Total Number of Citations of a Researcher
Total Years	Number of years since the academic's initial publication
Cites per Year	Total citations/years since first paper
Cites per Paper	Total citations/total papers
Author per Paper	Calculate the cumulative count of authors engaged in the publications attributed to the author in question, and subsequently divide this figure by the total number of papers.
Cites per author	Divide the citations for each individual publication by the corresponding number of authors, and then aggregate the resultant citation values. This sum represents the equivalent number of citations for the author in question as if they were the sole author.
Papers per author	Divide each publication by the number of authors and sum the fractional author counts; this is the single-authored equivalent number of papers for the author in question.
H-index	h = max (numbers of articles with >= h citations)
G-index	A collection of papers is said to possess a g-index of ‘g’ if ‘g’ represents the highest rank where the sum of citations from the top ‘g’ papers reaches or exceeds *g*^2^.
Hg-index	$hg-index=\sqrt{h⁎g}$ Where h represent h index and g represent g index.
A-index	$A-index=\frac{1}{h}\sum_{p=1}^{h}cit_{p}$ Where A is the A-index of the scholar, h represents the h-index and *cit*_*p*_ is the citation count of the *p*^*th*^ article.
R-index	$R-index=\sqrt{\sum_{p=1}^{h}Cit_{p}}$ Where h represents the h-index and *cit*_*p*_ is the citation count of the *p*^*th*^ article.
P-index	$P-index={\left(\frac{C^{2}}{p}\right)}^{\frac{1}{3}}$ The p-index strikes the best balance between total number of citations (C) and the mean citation rate (C/P)
Q^2^-index	$Q^{2}=\sqrt{h⁎m}$ Where h represent h index and m represent m index
K-index	$K-index=\frac{\frac{C}{P}}{\frac{C(h-tail)}{C(h-core)}}$ Where C represent total citation, p represents of *p*^*th*^ article, h-tail represent h-tail article citation and h-core represent h-core citation.
E-index	$e-index=\sum_{p=1}^{h}Cit_{p}-h^{2}$ Where *Cit*_*p*_ represent citation of *p*^*th*^ article and h represent h-index
f-index	$f-index=\left(\begin{array}{c} max\\ f \end{array}\right)\frac{1}{\frac{1}{f}\sum_{p=1}^{f}\frac{1}{Cit_{p}}}\geq f$ Where *cit*_*p*_ is the citation count of *p*^*th*^ article. The f-index never goes beyond the total number of publications.
T-index	$T-index=\left(\begin{array}{c} max\\ t \end{array}\right)exp\left[\frac{1}{t}\sum_{k=1}^{t}ln(Cit_{k})\right]\geq t$ Where *cit*_*k*_ is the citation count of *k*^*th*^ article
Tappered h-index	$H_{T(j)}=\frac{n_{j}}{2_{j}-1},n_{j}\leq j$ and $h_{T(j)}=\frac{j}{2_{j}-1}+\sum_{i=j+1}^{n_{j}}\frac{1}{2_{j}-1},n_{j}\geq j$
Wu index	The w-index of an author is determined by considering whether a minimum of ‘w’ of their articles have acquired ‘10w’ citations each, while the remaining publications have accrued fewer than ‘10(w+1)’ citations each.
Weighted h-index	$R_{w}(k)=\frac{\sum_{p=1}^{k}Cit_{p}}{h}$ Where h is the h-index and *cit*_*p*_ is the citation count of the *p*^*th*^ article. Then, the weighted h-index. Is defined as follows $h_{w}=\sqrt{\sum_{k=1}^{R}Cit_{k}}$ Where *cit*_*k*_ is the citation count of the *k*^*th*^ article and R is the largest rank among all publications such that the *k*^*th*^ weighted rank ≤ *cit*_*k*_.
h(2)-index	The h(2)-index is the maximum whole number such that the h(2) most cited articles by a scholar have each received at least (*h*(2))^2^ citations.
Woeginger index	$w=\left(\begin{array}{c} max\\ w \end{array}\right)(Cit_{p}\geq w-p+1)$ for all *p* ≤ *w* where *cit*_*p*_ is the citation count of *p*^*th*^ article and w is the maximum number of publications.
Rm-index	$R_{m}=\sqrt{\sum_{k=1}^{h}{\mathrm{Cit}}_{k}^{\frac{1}{2}}}$ Where *cit*_*k*_ is the citation count of *k*^*th*^ article.
m-index	An m-index is computed as the median quantity of citations garnered by the articles within the h-core.
X-index	$x=\sqrt{\left(\begin{array}{c} max\\ k \end{array}\right)kCit_{k}}$ Where *cit*_*k*_ is the citation count of *k*^*th*^ article.
h2 upper-index	$h^{2}upper=\frac{\sum_{k=1}^{h}(Cit_{k}-h)}{\sum_{k=1}^{m}Cit_{k}}⁎100=\frac{e^{2}}{\sum_{k=1}^{m}Cit_{k}}⁎100$ Where h is the h-index, *cit*_*k*_ is the citation count of the *k*^*th*^ article, *e*^2^ is the excess citation and m is the total number of articles.
h2 center-index	$h^{2}center=\frac{h⁎h}{\sum_{k=1}^{m}Cit_{k}}⁎100$ Where h is the h-index and *cit*_*k*_ is the citation count of the *k*^*th*^ article
h2 lower-index	$h^{2}lower=\frac{\sum_{k=h+1}^{m}(Cit_{k}-h)}{\sum_{k=1}^{m}Cit_{k}}⁎100$ Where h is the h-index and *cit*_*k*_ is the citation count of the *k*^*th*^ article
h^`^ –index (h dash)	$h^{\prime }=Rh=\frac{eh}{t}$ where R represents the head-tail ratio of e and t-index
Rational h-index	$h_{rat}=h+1-\frac{k}{2h+1}$ Where h is the h-index and k is the number of citations required to reach h+1 h-index value.
I10 index	This is a straightforward and direct indexing method that entails tallying the overall count of papers published by a journal, which have attained a minimum of 10 citations each.
Normalized h-index	$normalized\;h-index=\frac{h}{pub_{count}}$ Where h represents h index and pubcount represent total publication.
Π *index*	Π *index* = 0.01*C*(*P*Π) The Π − *index* is equal to the 100th of the number of citations, *C*(*Pπ*) to the top square root (*Pπ*) of the total papers(P).
Gh-index	$Gh^{a}=\sum_{p=1}^{m}sing(Cit({\mathrm{pub}}_{b}^{a})-GH)$ where *sing*(*x*)=1,*x* ≥ 0 and 0,*x* ≤ 0 Where m is the total number of publications of scholar a and GH is the h-index of the scholar. This index is also difficult to compute in comparison to the h-index.
W index	The w-index is characterized by the value ‘w’, denoting the count of an author's foremost articles that have accumulated no fewer than ‘10w’ citations individually. While the w-index can serve as a valuable metric for assessing a scholar's impact, it might impose a disadvantage on early-career researchers who have recently initiated their work or individuals who haven't yet produced a substantial volume of publications.
Maxprod	The maximum value of i * *c*_*i*_ can be found by evaluating the publication rank of a scholar, where *c*_*i*_ represents the number of citations for the *i*^*th*^ most frequently cited paper among all the citations.
H core citation	The h-core citation index exclusively considers publications that have been cited a minimum of ‘h’ times, disregarding those that haven't reached this threshold.
K dash index	$k^{\prime }=\frac{Cit_{all}-Pub_{Count}}{Cit_{T}-Cit_{H}}$ Where *cit*_*all*_ represent total citation, *pub*_*count*_ represent total publication, *cit*_*t*_ represent total citation of h tail article and *cit*_*h*_ represent total citation of h core article.
M-Quotient	$M-Quotient=\frac{h-index}{y}$ Where y represent no. of the year the first publication
Hc – index	$hc-index=\alpha ⁎\frac{C(i))}{\left(Y(now)-Y(i)+1\right)}$ Where Y(now) represent current year, Y(i) represent publication year, and C(i) represent paper i citation count. $hc-index=\frac{C(i)}{1},\frac{C(i)}{2},\frac{C(i)}{3}.........\frac{C(i)}{n}$
Aw-index	$Aw-index=\sqrt{\sum_{j=1}^{h}\frac{Cit_{j}}{a_{m}}}$ Where *Cit*_*j*_ citation of the article and *a*_*j*_ represent *a*^*th*^ article, m represent total article.
Ar index	$Ar-index=\sqrt{\sum_{j=1}^{h}\frac{Cit_{j}}{a_{j}}}$ Where *Cit*_*j*_ citation of the article and *a*_*j*_ represent *a*^*th*^ article, h represent total h core article.
AWCR	This parameter adjusts the citation count based on the length of time that has passed since each publication.
v-index	$V=\frac{h}{P(y_{this}-y_{o})}$ Where h is the h-index, *y*_*this*_ is the current year and *y*_0_ is the year of first publication.
Platinum H-index	$Platinium-h=\frac{H}{CL}⁎\frac{cit_{all}}{Pub_{count}}$ Where H is the h-index, CL is the career length, *Cit*_*all*_ is the total citation count and *pub*_*count*_ is the publication count.
Ha index	The ha-index against a dataset is the largest number of papers in the dataset that have obtained at least ha citations per year on average.
HI Index	$h_{i}=\frac{h}{Avg_{a}}$ where h represent h index and *Avg*_*a*_ represent average no. of authors in article.
hf index	$\frac{Yh_{f}}{\phi (Yh_{f})}\geq h_{f}$ where Y(i) represent citation count and *ϕ*(*i*) represent average no. of authors in article.
gf index	$gf=\sum_{{}^{i=1}}^{gf}\frac{Y_{i}}{\phi_{i}}\geq g^{2}f$ where Y(i) represent citation count and *ϕ*(*i*) represent average no. of authors in article.
gF index	$gF={\left(\sum_{i=1}^{k}\frac{1}{\phi (i)}\right)}^{2}\leq \sum_{i=1}^{k}y_{i}$ where Y(i) represent citation count and *ϕ*(*i*) represent average no. of authors in article.
Normalized Hi index	$\text{normalized hi index}=\frac{h}{pub_{count}}$ Where h represents the h-index and *pub*_*count*_ is the total number of articles.
Hm index	$r_{eff}(r)=\sum_{r^{\prime }=1}^{r}\frac{1}{a(r^{\prime })}\;then\;c(r(h_{m}))\geq h_{m}\geq c(r(h_{m})+1)$
k norm index	$k-norm=h-norm+(1-(h-\frac{norm^{2}}{\sum_{j=1}^{h-norm}citnorm_{i}}))$, ∨*h* − *norm* > 1 and *k* − *norm* = 0, if *h* − *norm* = 0
w norm index	$w-norm=h-norm+(1-(h-\frac{norm^{2}}{totalcit-norm})),\vee h-norm>0$ and $w-norm=\frac{totalcit-norm}{1+totalcit-norm}$, if *h* − *norm* = 0
gm index	*g*_*m*≤_*C*_*eff*_(*g*_*m*_) where *C*_*eff*_(*r*_*eff*_) and $S_{eff)}(r_{eff})=\sum_{r\overset{´}{=1}}^{r(r_{eff})}\frac{1}{a(r\overset{´}{})}c(r\overset{´}{})$
pure h index	$h_{p}(A)=\frac{h}{\sqrt{E(author)}}$ Where h represent h index and E average no. of author.
fractional h-index	$h_{f}=max(k\leq \frac{cit(k)}{author(k)})$ Where *Cit*_*k*_ represent citation of article and author(K) represent no. of author in a specific article.
fractional g-index	$g_{f}=max(\sum_{k=1}^{p}\frac{cit_{k}}{Author(k)}\geq p^{2})$
hi norm index	The hl-norm is a modified version of the h-index that normalizes citations based on the number of authors per paper.
K index	$Kindex=\frac{c/p}{c(h-tail)/c(h-core)}$ Where C represent total citation, P represent total publication, C(h-tail) represent total citation of h tail article and C(h-core) represent total citation of h core article.
Real h index	$h_{r}=\frac{(h+1)cit_{h}-h.cit_{h}+1}{1-cit_{h+1}+cit_{h}}$ Where h is the h-index and *cit*_*h*_ is the citation count of the *h*^*th*^ article.

## Data Availability

The dataset related to this study has been uploaded to a publicly accessible repository. The name of the repository is **ghulammustafacomsat** and the link is attached (https://github.com/ghulammustafacomsat/Mathematics_dataset).

## Footnotes

1 https://www.lms.ac.uk/.

2 https://www.mathunion.org/.

3 https://dnva.no/norwegian-academy-science-and-letters.

4 https://www.ams.org/home/page.

5 https://harzing.com/resources/publish-or-perish.
